# HERmione: Understanding the Needs of Patients Living with Metastatic HER2-Positive Breast Cancer Through a Cross-Sectional Survey in Parallel with Patients and Oncologists

**DOI:** 10.3390/cancers17081349

**Published:** 2025-04-17

**Authors:** Gilles Freyer, Caroline Rigault, Laure Guéroult Accolas, Anthony Barea, Narcisa Radu, Assia Ouamer, Mahasti Saghatchian

**Affiliations:** 1 CHU Lyon-Sud, Institut de Cancérologie et Université de Lyon, 69002 Lyon, France; 2 CHRU Tours, 37000 Tours, France; 3 Mon Réseau Cancer du Sein, 75006 Paris, France; 4 Wefight, 75013 Paris, France; 5 Seagen, 92500 Rueil-Malmaison, France; 6 Pfizer, 75014 Paris, France; 7 Hôpital Américain de Paris, Neuilly sur Seine, 92200 Neuilly-sur-Seine, France

**Keywords:** oncology, breast cancer, patient-reported outcomes, quality of life, clinician-reported outcomes, support needs, treatment preferences

## Abstract

Background/Objectives: HER2-positive metastatic breast cancer continues to have a significant impact on patients’ lives. The HERmione project was conducted in France to identify patients’ needs for support and information, understand services offered, and identify differences in the perception of burden between patients and oncologists. Methods: Between July and October 2022, 273 patients with HER2-positive metastatic breast cancer and 40 oncologists were surveyed. The mean age of patients was 52 years, with most receiving treatment at specialized cancer centers (38%) or other public hospitals (34%). Results: The survey revealed a substantial burden of the disease and treatment in patients, perceptions that differed from those of oncologists. Both the physical and mental well-being of the patients were below average. Despite the burden of the disease, patients lacked access to many types of support, particularly support with sexual well-being. Additionally, 60% of patients did not have access to nursing support. Patients had high expectations regarding access to information but often did not know where to access this information. Despite this, they still exhibited treatment preferences. Conclusions: These findings suggest that enhanced communication is critical to ensure that patients receive adequate support. Nursing support could improve patient–oncologist communication and thereby enhance patient well-being. Finally, to meet patient expectations regarding information access, a broader array of support tools should be offered.

## Simple Summary

The aim of this study was to survey patients with HER2-positive metastatic breast cancer and oncologists to identify the difficulties experienced by patients and compare their support needs with the healthcare services available. Overall, 273 patients responded, with a mean age of 52 years. Patients reported substantial burdens from both the disease and its treatment, which were often underestimated by oncologists. They also had lower physical and mental well-being than average. Patients did not have access to many types of support, especially for sexual well-being, and about 60% lacked nursing support. Patients often did not know where to find the information they needed. This study suggests that better communication is needed to ensure that patients receive the necessary support, which could be improved with better nursing support and a wider range of informational tools. Overall, these measures should enhance patient well-being.

## 1. Introduction

Breast cancer is the most commonly diagnosed cancer in women, accounting for 13% of all new cancer cases worldwide in 2020 [1]. Between 13% and 15% of breast cancers overexpress human epidermal growth factor receptor 2 (HER2) [2,3,4,5]. Historically, HER2-positive breast cancer has been associated with a more aggressive phenotype and worse prognosis than other subtypes [2,6,7]; however, HER2-targeted treatments have improved survival rates and reduced the risk of relapse in early-stage disease [8,9,10]. In metastatic HER2-positive breast cancer, combination therapy with chemotherapy and HER2-targeting agents has extended median overall survival to 4.5 years, compared to 1.5 years with chemotherapy alone [11,12]. Despite these therapeutic innovations improving disease prognosis and life expectancy, the disease continues to have a significant impact on patients’ lives. It is still considered incurable, with many patients eventually developing resistance to treatment [13].

In the last decade, Patient-reported Outcomes (PROs) have been increasingly considered as an important tool to improve treatment and care decisions, particularly in palliative care, as, in these cases, treatment should not aim only to extend life but should also aim to improve patients’ quality of life (QoL) [14]. Indeed, PROs allow for a direct report from the patient about symptoms not directly observable by the clinicians and help better understand the impact of the disease beyond treatment [15]. In addition to a better tailoring of treatment options to patients’ needs, it increases patient involvement in their treatment options, leading to heightened levels of satisfaction in healthcare and better observance [16].

As diseases can have specific symptoms, treatment options and outcomes will therefore impact patients’ life in different and specific ways, making it important to have tailored PROs that best align with the patient experience. A study gathering data from three clinical trials showed that PROs can be a prognosis for overall survival [17], but there is limited data examining PROs from patients with metastatic HER2-positive breast cancer, which includes the patients’ needs and the impact of the disease on their QoL. Additionally, the therapeutic landscape is in constant evolution, leading to rapidly evolving therapies and management strategies. This in turn makes non-treatment-related PROs especially relevant, as they cover all potential adverse effects, regardless of treatment, and can continue to be used regardless of future evolutions in the therapeutic strategies. In particular, and despite the recognized need for a strong communication between patients and clinicians, surveys conducted in parallel, to compare PROs with clinician-reported outcomes (ClinROs), are underutilized or under-published.

The HERmione project, conducted in France, was carried out by Centre Hospitalier Régional Universitaire Tours, Hospices Civil de Lyon, Hôpital Américain de Paris, Wefight, Seagen, which was acquired by Pfizer in December 2023, and Mon Réseau Cancer du Sein. Consequently, the pioneering HERmione survey was designed to tackle this gap in information and explore the situation in France. The project applied a multistakeholder recruitment approach to reach and run a survey in parallel, with both populations of patients and oncologists in France.

The QoL component of the survey was based on the SF-12v2^®^ PRO Health Survey (SF-12), a PRO tool that measures the impact of symptoms and diseases across eight health domains, with 12 questions aimed to improve the understanding of patient experience [18,19].

The HERmione survey aimed to identify and prioritize patients’ needs for support and information; better understand their experiences and perceptions of treatment and healthcare services offered to patients; and identify potential differences in perception between patients and oncologists regarding their experience of the disease and its management.

## 2. Materials and Methods

### 2.1. Survey Design

This cross-sectional study was designed to assess the views of patients and oncologists using mirrored surveys to obtain a crossed-view perspective. The structure of the two surveys was similar but not identical.

### 2.2. Patient Recruitment

Patients with HER2-positive metastatic breast cancer were recruited in France through the Wefight network using the Vik Breast app, a Chatbot designed to interact and support patients [20], the Mon Réseau Cancer du Sein Association, and healthcare professionals on the Steering Committee. To participate, patients had to be female, older than 18 years old, and diagnosed with HER2-positive metastatic breast cancer. Patients who were not diagnosed with HER2-positive metastatic cancer were not included in this study. The survey was conducted between 22 July and 6 October 2022.

### 2.3. Patient Survey Design

Patients were invited to take part in an anonymous 42-question survey. Those recruited through the Wefight network and the Mon Réseau Cancer du Sein Association completed the survey online, while those recruited through the Steering Committee were given hard copies of the survey at three hospitals (Lyon, Paris, and Tours). The full survey is provided in Supplementary File S1. All clinical characteristics of the patients were self-reported and derived from responses to the study questionnaire. Quality of life was assessed using the SF-12 questionnaire, a standardized PRO measure.

### 2.4. Oncologist Recruitment

A representative sample of oncologists caring for patients with metastatic HER2-positive breast cancer were recruited in France, amongst a panel of healthcare professionals established by the Wefight network. Representativeness was ensured through the quota method recommended by the French Directorate for Research, Studies, Evaluation, and Statistics, and applied to variables of gender, age, and practice setting. Oncologists who did not treat patients with metastatic HER2-positive breast cancer were not included in this study. The survey was conducted between 25 June and 12 September 2022.

### 2.5. Oncologist Survey Design

Oncologists took part in an anonymous 26-question survey to assess their parallel views on the same topics as patients. The full survey is provided in Supplementary File S2.

### 2.6. Data Analysis

Data were only analyzed from participants who completed the full survey. Responses from those under 18 years of age were excluded from analysis. Any surveys that were completed in under 5 min were excluded from the analysis for quality control purposes. Open-ended responses were analyzed using a hybrid approach combining inductive and deductive coding. Initially, two researchers independently reviewed answers to identify emerging themes. A preliminary codebook was then developed based on these themes. The full dataset was coded accordingly, with discrepancies resolved through discussion to ensure consistency.

### 2.7. Statistical Analysis

Statistical analyses were mainly descriptive. However, patients’ and oncologists’ responses were compared using chi-squared tests or Fisher’s exact tests, whenever appropriate. The significance threshold was set at a 5% level. No adjustment for multiplicity was performed.

### 2.8. Ethics

As this was a non-interventional study, no ethical review was required. Consent to share data was embedded in the survey.

## 3. Results

### 3.1. Patient Demographics

A total of 273 patients completed the survey: 268 online and 5 using hard copies. All patients were female, and the mean age was 52 years, with most falling into the 45–54 (39%) age range (Table 1). Most patients already had metastatic cancer on diagnosis (55%), had been diagnosed in the last five years (61%), and were being treated at a specialized cancer center (38%) or other public hospitals (34%). The majority were not professionally active (54%).

Only 36 (13%) patients were aged 65 years or over, and, so, differences between these and younger patients have only been stated where significant. These patients were more likely to present with metastatic cancer on diagnosis (72%) and were more likely to be retired (94%).

### 3.2. Oncologist Demographics

Of the 40 oncologists who completed the survey, most were male (65%). The mean age of oncologists was 42 years, with most falling into the under 40 years (52%) category (Table 2). Most oncologists worked in hospital centers (65%) and spent a mean of 33% of their activity treating HER2-positive patients. On average, half of the patients treated in their clinic with HER2-positive breast cancer had metastatic disease.

### 3.3. Burdensome Side Effects of Treatment and Symptoms of the Disease

To assess the impact of treatment burden, patients were asked, “which side effects of your current treatment are the most difficult to manage on a daily basis?” To assess their parallel views on the topic, oncologists were asked, “in your opinion, what are the most difficult side effects related to treatments for patients with metastatic HER2-positive cancer to manage?”

**Figure 1:**
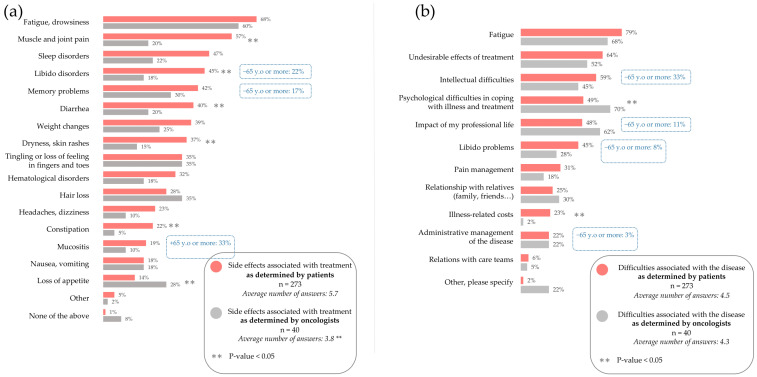
The burdensome effects of treatment and HER2-positive breast cancer itself as determined by patients and oncologists: (**a**) Patients are asked, “which side effects of your current treatment are the most difficult to manage on a daily basis?”; oncologists are asked, “in your opinion, what are the most difficult side effects related to treatments for patients with metastatic HER2-positive cancer to manage?”. (**b**) Patients are asked, “what are the main difficulties that you have encountered since the announcement of your disease at the metastatic stage?”; oncologists are asked, “in your opinion, what are the main difficulties that patients encounter since the announcement of their disease at the metastatic stage?”. Patients, n = 273. Oncologists, n = 40. 65+, n = 36. Percentages do not add to 100% as multiple answers could be selected.

The three most burdensome side effects reported by patients were fatigue (68%), muscle and joint pain (57%), and sleep disturbances (47%) (Figure 1a). While oncologists also considered fatigue to be the most burdensome side effect (60%), they significantly underestimated the burden of muscle and joint pain (20%, *p*-value < 0.001) and sleeping troubles (22%, *p*-value = 0.007) (Figure 1a). Instead, oncologists considered hair loss and tingling or loss of sensitivity in fingers and toes to be the second most burdensome side effects.

Oncologists significantly underestimated the burden of several more side effects, including troubles with libido (45% of patients vs. 18% of oncologists, *p*-value = 0.002), skin dryness and rashes (37% of patients vs. 15% of oncologists, *p*-value = 0.009), and diarrhea (40% of patients vs. 20% of oncologists, *p*-value = 0.027). Additionally, oncologists reported significantly fewer side effects on average (3.8) than patients (5.7).

Patients aged 65 and over reported a significantly lower burden of libido troubles (17%, *p*-value = 0.001) and memory disorders (22%, *p*-value = 0.014) than the general population (Figure 1a).

Patients were next asked specifically about the burden of their disease, rather than treatment side effects. Similarly, oncologists were also asked their perspectives on the burden of the disease experienced by patients.

The three most encountered disease-related difficulties reported by patients were fatigue (79%), undesirable effects of treatment (64%), and intellectual difficulties (59%) (Figure 1b). While oncologists did not significantly underestimate the burden of these difficulties (68%, 52%, and 45%, respectively), they did consider psychological difficulties a greater burden than all three (70%) (Figure 1b). This was significantly (*p*-value = 0.019) overestimated compared to patients (49%). Oncologists also significantly underestimated the patients’ burden of financial costs (23% vs. 2%).

Patients aged 65 years and over report a significantly lower burden of disease on intellectual difficulties (33% vs. 59%, *p*-value = 0.001), libido troubles (8% vs. 45%, *p*-value = 0.001), and administrative management (3% vs. 22%) than the general patient population (Figure 1b).

### 3.4. Patient Quality of Life and Well-Being

To assess patients’ positive and negative feelings regarding their illness, patients were asked to choose the feelings that applied to them from a list. To assess oncologists’ perceptions of patients’ feelings, oncologists were asked to choose the feelings that they thought applied to their patients.

**Figure 2:**
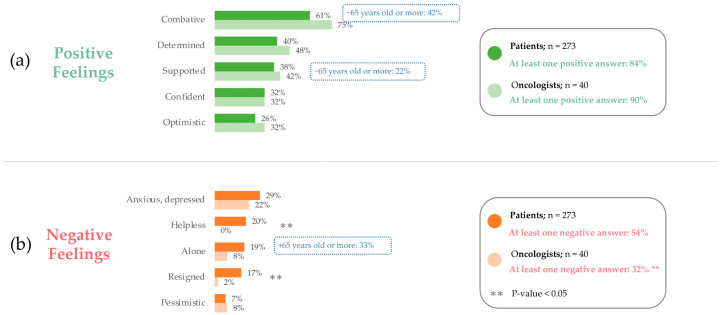
Positive and negative feelings reported by patients and oncologists: (**a**) Patients are asked, “would you say that regarding your illness, you currently feel…”. (**b**) Oncologists are asked, “would you say that regarding their illness, patients with HER2-positive cancer especially feel…”. Patients, n = 273. Oncologists, n = 40. 65+, n = 36. Percentages do not add to 100% as multiple answers could be selected.

Most patients (84%, Figure 2a) and oncologists (90%, Figure 2b) reported at least one positive feeling. There was no significant difference in reported positive feelings between patients and oncologists. The most common positive feeling was “combative” (having a fighting mindset), reported by 61% of patients and 75% of oncologists. Many patients also reported feeling “determined” (40%) and “supported” (38%), compared to 48% and 42% of oncologists, respectively. Compared to the general patient population, significantly fewer patients aged 65 and over reported feeling “combative” (42%, *p*-value = 0.019) and “supported” (22%, *p*-value = 0.049) (Figure 2a,b).

Regarding negative feelings, significantly fewer oncologists (32%) reported at least one negative feeling compared with patients (54%), indicating that they underestimate the negative feelings of patients. In both groups, the most reported negative feeling was “anxious/depressed,” reported by 29% of patients and 22% of oncologists. “Helplessness” was the second most reported negative feeling by patients (21%) yet was not reported by any oncologists (0%). Oncologists also significantly underestimated the patients feeling “helpless” (20% of patients vs. 0% of oncologists, *p*-value = 0.004) and “resigned” (17% vs. 2%, *p*-value = 0.033). Compared to the general population, a significantly greater number of patients aged 65 and over reported feeling “alone” (33%, *p*-value = 0.034).

**Figure 3:**
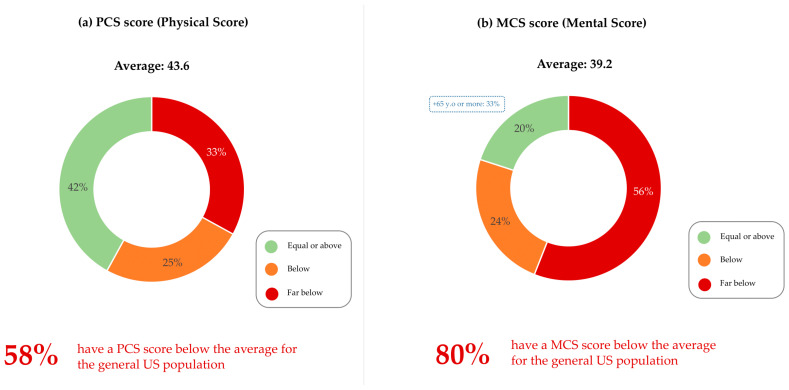
Quality of life of patients as assessed by the SF-12 questionnaire: (**a**) Mean Physical Component Score (PCS) of patients and percentage below or above the US national average of 50. (**b**) Mean Mental Component Score (MCS) of patients and percentage below or above the US national average of 50. All patients, n = 273. 65+, n = 36.

Next, QoL was assessed using the SF-12 questionnaire, a PRO measure. The final score includes a Physical Component Summary (PCS) and Mental Component Summary (MCS) and is measured on a scale of 0 to 100. Both the PCS (43.6) and the MCS (39.2) were considerably lower than the US national average of 50. Regarding the PCS, 58% of patients were below the average of the general population, 33% of whom were much below (Figure 3a). A total of 80% of patients were below average in the MCS, including 56% much below (Figure 3b).

Patients aged 65 and over exhibited no significant difference in PCS and MCS scores (Figure 3a,b).

### 3.5. Support Available to Patients

**Figure 4:**
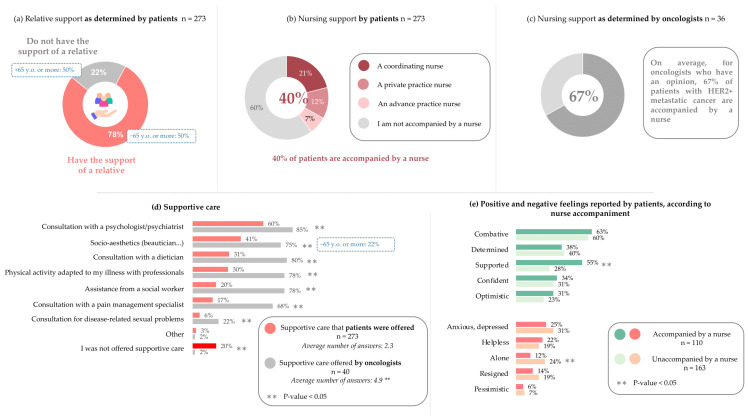
Support available to patients as determined by both patients and oncologists: (**a**) Patients are asked, “do you have the support of a relative?”. (**b**) Patients are asked, “As part of your follow-up in a health establishment, in addition to your oncologist, are you accompanied by a…”. (**c**) Oncologists are asked, “In your opinion, as part of their follow-up, what percentage of patients with metastatic HER2-positive cancer are accompanied by a nurse (coordination, advanced practice, private)?”. (**d**) Patients are asked, “have you been offered one or more of the following supportive care since you were diagnosed with metastatic disease?”; oncologists are asked, “do you offer the following different supportive care to your patients with metastatic HER2-positive cancer?”. (**e**) Patients are asked, “would you say that regarding your illness, you currently feel…”, and their answers are grouped based on whether they are supported by a nurse. Patients, n = 273. Oncologists, n = 40. 65+, n = 36. Supported by a nurse, n = 110. Not supported by a nurse, n = 163. For panels d and e, percentages do not add to 100% as multiple answers could be selected.

We next wished to evaluate the level of support available to patients. When patients were asked about their support network, 78% reported that they had the support of a relative, while 22% did not (Figure 4a). Significantly fewer patients aged 65 and over had the support of a relative (50%, *p*-value < 0.001).

Next, patients were asked about the supportive care received since diagnosis of metastatic disease. In total, 80% of patients reported that some type of support had been offered to them. On average, patients reported that 2.3 types of care had been offered to them (Figure 4d). When oncologists were asked about types of care offered, they reported offering 4.9 types of care on average, a significant overestimation compared to that reported by patients (Figure 4d). Additionally, oncologists significantly overestimated the amount of support offered for every type of care, including psychological support (60% of patients vs. 85% of oncologists, *p*-value = 0.005), socio-aesthetic support (41% vs. 75%, *p*-value < 0.001), nutritional support (31% vs. 80%, *p*-value < 0.001), adapted physical therapy (30% vs. 78%, *p*-value < 0.001), social worker (20% vs. 78%, *p*-value < 0.001), pain management (17% vs. 68%, *p*-value < 0.001), and support with sexual well-being (6% vs. 22%, *p*-value = 0.002). Patients aged 65 and over were offered significantly less socio-aesthetic support (22%, *p*-value = 0.025) (Figure 4d). They also reported less support in all other areas, albeit not significantly, except pain management.

To assess patients’ access to nursing support, patients were asked whether they were accompanied by a nurse, of whom 40% reported that they were (Figure 4b). To obtain a crossed-view perspective, oncologists were asked what percentage of patients with metastatic HER2-positive cancer they believe are accompanied by a nurse. Oncologists overestimated nursing support, estimating that 67% of patients are accompanied by a nurse (Figure 4c).

To determine whether nursing support is beneficial to patients, the positive and negative feelings reported by patients were grouped based on whether the patient reported feeling accompanied by a nurse. Nursing support was associated with feeling significantly more “supported” than those without nursing support (55% vs. 28%, respectively, *p*-value < 0.001), as well as feeling significantly less “alone” (12% vs. 24%, *p*-value = 0.019) (Figure 4e). Additionally, significantly more patients who were accompanied by a nurse felt that it was easy to call on the healthcare team when needed, compared to those not accompanied by a nurse (89% vs. 69%, *p*-value < 0.001).

**Figure 5:**
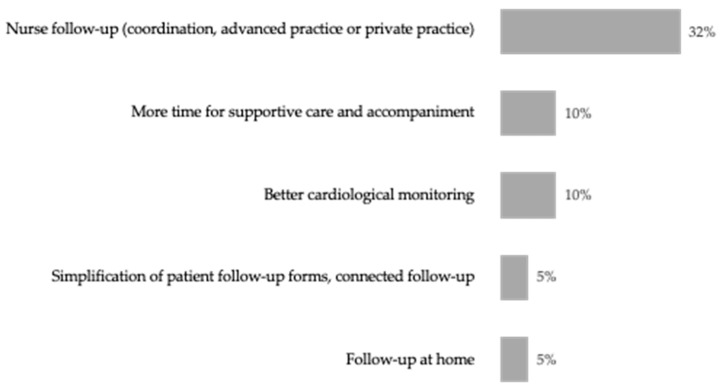
Oncologists’ top five suggestions on how to improve the journey of patients. Oncologists are asked, “in your opinion, what could be implemented to improve the journey of patients with metastatic HER2-positive breast cancer within your establishment?”. Only the top five responses are shown. n = 40. Percentages do not add to 100% as multiple answers could be selected.

When oncologists were asked “in your opinion, what could be implemented to improve the journey of patients with metastatic HER2-positive breast cancer within your establishment?”, almost one-third (32%) suggested nursing support, compared to all other suggestions that were mentioned by less than 10% of oncologists (Figure 5).

### 3.6. Patient Treatment Preferences and Engagement in Choosing a Treatment

**Figure 6:**
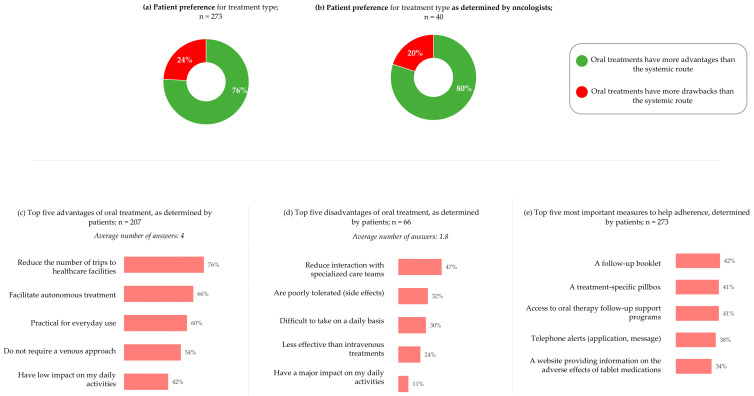
Preferences for oral treatment, perceived advantages and disadvantages, and measures to improve adherence at home: (**a**) Patients are asked, “in your opinion, the use of oral treatments (capsule/tablet) specific to your metastatic breast cancer presents…”. (**b**) Oncologists are asked, “in your opinion, for patients, the use of targeted therapies, oral anti-cancer treatments specific to these metastatic HER2-positive breast cancers present…”. (**c**) Patients are asked, “what do you think are the main advantages of using oral treatments (capsule/tablet) specific to your breast cancer?”. Only the top five responses are shown. (**d**) Patients are asked, “what do you think are the main disadvantages of using oral treatments (capsule/tablet) specific to your breast cancer?”. Only the top five responses are shown. (**e**) Patients are asked, “in your opinion, what would be important to put in place to help patients take anti-cancer treatment in oral form (tablet/capsule) at home?”. Only the top five responses are shown. Patients, n = 273. Oncologists, n = 40. For panels c, d, and e, percentages do not add to 100% as multiple answers could be selected.

We next wished to determine patient treatment preferences and whether these were considered by oncologists when prescribing. When asked about their preferences concerning oral and systemic treatment, both groups agreed that oral treatment is preferable to systemic treatment (76% of patients and 80% of oncologists) (Figure 6a,b). The top five advantages of oral treatment stated by patients were as follows: less need to travel to healthcare facilities (76%), autonomy in treatment (66%), practicality in daily life (60%), does not require venous infusion (54%), and positive impact on daily life (42%) (Figure 6c). Conversely, the top five disadvantages were loss of access to specialized care team (47%), poor tolerance/side effects (32%), difficulty in taking treatment daily (30%), less effective than systemic treatment (24%), and negative impact on daily life (11%) (Figure 6d).

Patients were then asked which measures they thought would be important to help them take oral anti-cancer medications at home. The top five most reported suggestions were a follow-up diary (42%), a pillbox (41%), follow-up support programs (41%), telephone reminders (38%), and a website on the unwanted side effects of oral therapy (34%) (Figure 6e).

**Figure 7:**
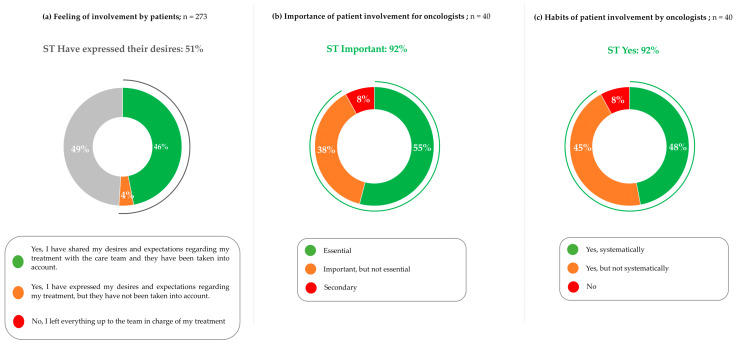
Patient involvement in the treatment decision, as determined by patients and oncologists: (**a**) Patients are asked, “did you feel that your opinion was taken into account when initiating the last treatment you received?”. (**b**) Oncologists are asked, “in your opinion, involving patients in the choice of treatments is…”. (**c**) Oncologists are asked, “Would you say that you involve patients in the choice of treatments at the time of initiation?”. Patients, n = 273. Oncologists, n = 40. Percentages may not add to 100% due to rounding.

When patients were asked “did you have the feeling that your opinion was taken into account when initiating the last treatment you received?”, 46% reported that they expressed their wishes and expectations to the healthcare team, which were taken into account, 4% reported that their wishes and expectations were not taken into account, and 49% reported that they left the decision to the healthcare team (Figure 7a).

Most oncologists considered patient involvement in treatment choice to be “essential” (55%) or “important but not essential” (38%) (Figure 7b). When asked about whether they involve patients in treatment choices 48% answered “yes, systematically,” 45% answered “yes, but not systematically”, and 8% answered “no” (Figure 7c).

### 3.7. Patient Access to Information on Research and New Treatments

**Figure 8:**
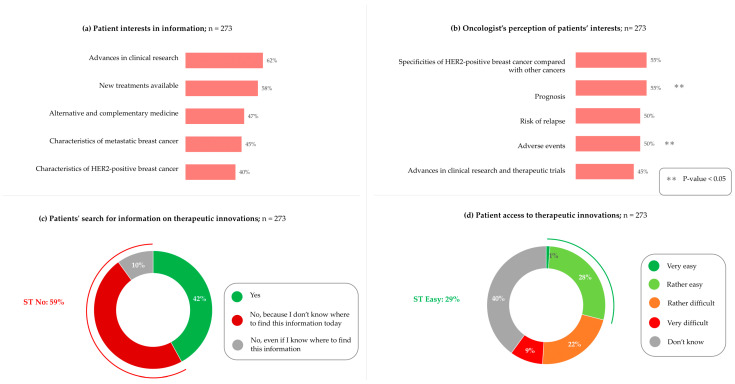
Information interests as determined by patients and oncologists, and access to required information: (**a**) Patients are asked, “which of the following aspects of your illness do you need information about?”. Only the top five responses are shown. (**b**) Oncologists are asked, “which of the following aspects of metastatic HER2-positive cancer do your patients need information about?”. Only the top five responses are shown. (**c**) Patients are asked, “are you finding out about innovations in treatments for HER2-positive breast cancer?”. (**d**) Patients are asked, “In your opinion, access to these innovative treatments for patients in France is…”. Patients, n = 273. Oncologists, n = 40. For panels (**a**,**b**), percentages do not add to 100% as multiple answers could be selected. For panels c and d, percentages may not add to 100% due to rounding.

Finally, we wished to assess the information needs of patients. When patients were asked which aspects of their illness they need information about, they expressed most interest in information about advances in clinical research (62%) and new treatments available (58%) (Figure 8a). Patients also expressed high levels of interest in other types of information, including alternative and complementary medicines (47%), as well as information on metastatic disease (45%) and HER2-positive breast cancer (40%). When oncologists were asked which information they believed that patients needed, they significantly underestimated their need for information on new treatments (30%, *p*-value = 0.001) and metastatic disease (20%, *p*-value = 0.004) (Figure 8b).

When patients were asked about whether they are seeking information, 49% stated that they did not know where to find the information they require (Figure 8c). Additionally, 31% found that access to information on new treatments was rather difficult or very difficult, while 40% did not know (Figure 8d).

## 4. Discussion

The unmet needs of breast cancer patients have previously been examined using PROs, with these unmet needs affecting both physical and mental QoL [21,22]. However, HER2-positive breast cancer generally requires different treatment to other subtypes [23]**,** and patients may therefore experience different effects on QoL and have different support needs. The aim of this survey was to assess the burden of HER2-positive breast cancer on the QoL of patients, both PROs and ClinROs. Additionally, we wished to evaluate discrepancies between the support requirements of patients and access to required support, including nursing support. This would allow the identification of current strengths and areas that warrant further development in patient support and ultimately help to build recommendations to improve patient care and QoL.

From this survey, we have found that HER2-positive patients report difficulties with treatment including fatigue, muscular and joint pain, and difficulty sleeping. When asked the same question, oncologists tended to indicate fewer concerns, underestimating many of the most important challenges to patients. These results are aligned with a previous study that described differences in the burden reported by patients compared to the perceived burden by healthcare providers [24]. Similarly to the oncologists asked in our survey, healthcare providers underestimated the burden of sleeping difficulties [24]. This suggests that communication between patients and oncologists could be improved when discussing QoL and that oncologists could consider both ClinROs and PROs when discussing QoL with patients.

Patients reported several side effects that are likely to compound to the detriment of their sexuality, including the loss of libido. Skin dryness and troubles may also contribute, given that this may include patients suffering from vaginal dryness, a common side effect of breast cancer treatments [25]. Additionally, hair loss and weight loss were reported in a considerable amount of patients, both of which have been previously associated with body image, which in turn are associated with sexual problems [25].

Despite the burden of the disease on sexuality, oncologists identified other burdens of higher priority, meaning that patients’ need for support with sexual well-being is partly unmet. For example, while 60% of patients were offered psychological support, only 6% were offered support for sexuality even though 45% of patients report problems with their libido. Discrepancies in perceived burden may arise from difficulties in communication, which could be improved through systematic enquiry about the full range of potential difficulties. However, discussing the full range of difficulties is likely to require a longer consultation time and could also require extra resources, such as appointments with specialized nurses. Some associations are already active in this field, for example, AFSOS (https://www.afsos.org/ (accessed on 25 January 2024)) (Association Francophone des Soins Oncologiques de Support), a French association promoting the awareness and implementation of recommendations for supportive care. They provide patient friendly factsheets with key information and advice on topics such as psychological support, self-care, and feeling self-confident, as well as providing a link to identify patient associations proposing supportive care in their regions (La vie autour, initiative sponsored by AFSOS and Pfizer). Interestingly, sexuality is not a topic where supportive care can be easily identified as it is not part of the search criteria on the La vie autour (https://www.lavieautour.fr/ (accessed on 25 January 2024)) website.

Our survey also reported the positive and negative feelings of patients. Despite feeling generally combative and determined, they nonetheless suffer from feelings of anxiety, depression, helplessness, and loneliness. Additionally, the physical and mental well-being of patients are both below average, collectively indicating that living with their disease brings a range of psychological challenges. This is recognized as a top priority by oncologists, who often offer psychological support to patients.

The observation that patients report lower-than-average well-being scores and often report psychological difficulties is expected given that the psychological burden of cancer has been well documented. The prevalence of depression and anxiety in the general population are estimated to be around 5% and 7%, respectively [26]. However, the prevalence of major depression in patients treated for cancer has been estimated at around 15%, with anxiety estimated at around 10% [27]. Additionally, patients with cancer are at an almost two-fold greater risk of dying by suicide than the general population [28]. Breast cancer ranked seventh for the cumulative burden of five psychiatric disorders (depression, anxiety, schizophrenia, bipolar disorders, and personality disorders), demonstrating the impact of breast cancer, in particular, on psychological well-being [29].

Perhaps more unexpected is the overestimation of psychological difficulties by oncologists. However, as previously mentioned, discrepancies between patients and healthcare providers have previously been reported. While the paper did not examine psychological difficulties, it did report overestimations by healthcare providers of the impact of cancer on several areas [24].

The overestimation of the burden of cancer on psychological difficulties combined with underestimations on fatigue, sleeping difficulties, muscle and joint pain, and libido suggest a scope for exploring ways to develop and improve the exchanges of communication and understanding between patient and oncologist.

Communication can be facilitated through nursing support, which is a valuable tool to ensure that patients have access to the information and advice needed to support them through their diagnosis and treatment choices [30]. Nurses are also able to provide important psychosocial support [31], promote adherence to therapy by ensuring that patients understand the necessity of treatment [32], and build close relationships between patient and care services [30].

Around one-third of oncologists recommended that nursing support be added as a means of improving patient care pathways, indicating that they value this service to patients. Despite this, nursing support is not systematic. Only 40% of patients reported being accompanied or supported by a nurse during their follow-up. A comparative analysis showed that patients supported by a nurse feel significantly more surrounded in dealing with the disease and are more likely to feel that it is easy to reach out to the healthcare team. Extending nursing care to all patients would therefore likely have a beneficial effect on patient well-being. Effective nursing support, particularly support that can be reached when the patient requires, may give the patient more opportunity to raise any concerns.

Additionally, contact with nurses, whether in person or remotely, may give patients the information and support needed to prepare for appointments with their oncologists. Such preparation can help to bridge the gap in communication, to help better align both parties’ perceptions of the burden of cancer.

Patients express high expectations in terms of access to information about their disease and about therapeutic innovation. This is supported by previous findings that access to information for breast cancer patients is rated as 4.15 on a 5-point scale, with 1 being not important and 5 being extremely important [33]. The perceived importance did not change between years 1, 3, and 5 following diagnosis, highlighting the need for ongoing support [33]. Additionally, patients require information on a range of themes, including etiology, diagnosis, treatment, prognosis, scientific research, and impact on normal life [34].

In the first few months post-diagnosis, patients rely on their healthcare provider as their primary source of information [35]. Patients with lower access to information have a lower perception of health competence and lower emotional, functional, and social well-being [36]. Conversely, those receiving adequate information from their clinician are more likely to feel satisfied with their treatment [37]. Additionally, patients with better access to information feel more comfortable in weighing up the benefits and risks of treatment and subsequently making treatment decisions [38]. Access to information is therefore important, not only to support a patient through their diagnosis but also to help them understand their treatment options and establish their individual preferences regarding treatment.

In our survey, over three-quarters of patients preferred the oral route to other routes of treatment. These patients felt that oral treatments prevent numerous trips to healthcare facilities, facilitate autonomy in treatment, and are convenient in daily life. The main disadvantage associated with these treatments is the reduction in interaction with the healthcare team. Most oncologists agreed that the oral route of treatment was preferable, suggesting that they are likely to prescribe oral treatments where possible.

The vast majority of oncologists indicated that they were in favor of patients taking part in the treatment choice, although only around one-half report doing so systematically. This indicates an area in which patients can increase their engagement. It has previously been reported that most patients prefer to contribute to the treatment decision, but physicians are often not aware of this [39]. Without this awareness, patients may not consistently be asked about their preferences. To provide a patient-centered treatment experience, the treatment decision should always be shared between patients and oncologists, including the method of administration and convenience.

Our research reveals discrepancies in how patients and oncologists perceive the burden of the disease. It also highlights a shortfall in provided support, information access, and patient involvement in treatment decisions. This signals a clear communication gap throughout diagnosis and treatment. Effective communication between patients and healthcare professionals is an essential factor to ensure patient well-being and could be facilitated by extending access to nursing support to all patients.

To summarize, the HERmione project survey provides several key differentiating facts compared to other similar surveys on HER2-positive metastatic breast cancer. It highlights the existence of a patient–oncologist perception gap, with a significant difference in the perception of disease burden between patients and oncologists. This gap is not always emphasized in other surveys, which often focus more on clinical outcomes rather than perceptual differences [17,40]. Another notable finding is the lack of access to various types of support, especially sexual well-being support and nursing support. This specific focus on sexual well-being is less commonly addressed in other surveys. The survey also underscores that patients have high expectations for information access but often do not know where to find it. This specific issue of information accessibility and patient expectations is a unique aspect of the HERmione project. The lack of nursing support for 60% of patients is a critical finding. While other surveys may mention general support needs, the specific emphasis on nursing support and its potential to improve patient–oncologist communication is a distinguishing factor. Finally, this survey reveals that patients still exhibit treatment preferences despite information gaps. This indicates a proactive approach by patients in managing their treatment, which is not always captured in other surveys.

These differentiating facts suggest that the HERmione project provides a more nuanced understanding of the patient experience, particularly in terms of support needs and the communication gap between patients and oncologists

This survey is limited by the relatively small sample size of oncologists, which may introduce bias. Additionally, of the total number of patients that took the survey, only 13% were aged 65 and over, making it challenging to draw meaningful conclusions relating to this sub-population. The bias towards younger patients may be attributed to the use of an app for data collection, and, therefore, in future studies, a greater effort should be made to collect data using hard copies of the survey. While the survey provides insights into the national landscape, caution should be taken when applying findings to other countries, given the differing systems of social support and healthcare. Similarly, conclusions from this survey may not be applicable to other types of cancer, or even other types of breast cancer, due to the diverse treatments and types of support offered. Finally, although PROs offer valuable insights into patient experience, factors such as subjectivity, the individual’s response style, and patient comfort levels with the setting and mode of administration may influence the reliability of patient reporting [41].

## 5. Conclusions

For the first time, the patient association group Mon Réseau Cancer du Sein built a unique collaboration with a scientific committee and Pfizer to present a groundbreaking study about patients living with HER2+ metastatic breast cancer, to explore the impact of this disease on patients’ quality of life. HERmione has given a voice to 273 patients and 40 oncologists, and this unprecedented cross-sectional study has unveiled new insights on the experiences and preferences of patients living with HER2+ metastatic breast cancer. To our knowledge, in the modern era of Her2 overexpressing breast cancer, this study is the most exhaustive one of its category. In two decades, the prognosis of this tumor subtype has been dramatically improved, and patients with Her2+ breast cancer, notably HR positive tumors, currently have the best prognosis of all breast cancer subtypes. Most patients can be cured at an early stage, while median overall survival in the metastatic setting is around 60 months. For a majority of patients with metastases, the disease has become chronic and deserves a holistic approach, as asked by the patients. The exploration of the various dimensions of the supportive care in this setting, including PROs, is probably the main originality of this study.

Indeed, our survey sheds light on numerous unmet needs among those patients. One notable finding is the limited availability of comprehensive support options, such as support with sexual well-being, which can significantly impact patients’ well-being. Access to nursing care emerges as a crucial factor in providing patients with a sense of support and mitigating feelings of isolation; however, a significant portion of patients lack access to this essential resource. Furthermore, our study highlights the high expectations that patients have regarding access to information, despite facing challenges in locating reliable sources. Nonetheless, patients are able to express treatment preferences, particularly favoring oral treatments due to the autonomy they afford.

These results have allowed the identification of new ways to further improve the care of these patients and, above all, fueled new reflections on the solutions to be deployed to improve patients’ daily lives, leading to the implementation of additional actions.

In conclusion, better communication is essential to determine the type of support required to improve patient care. This could be facilitated by combining ClinROs with PRO measures, such as symptom questionnaires, as well as ensuring access to nursing support to bridge the gap in patient–oncologist communication and improve patient well-being. Patient preferences for treatment may be influenced by many considerations; therefore, greater patient involvement in therapy choice should be given. Finally, to meet patient expectations regarding information access, a broader array of support tools could be offered, both digital and in-person.

## Figures and Tables

**Table 1:** Patient demographics and disease characteristics (N = 273).

Variable	N (%)
Age	
18–44 years	62 (22.7%)
45–54 years	106 (38.8%)
55–64 years	69 (25.3%)
65 years and over	36 (13.2%)
Missing	0
Professional activity	
Inactive	147 (53.8%)
Active	78 (28.6%)
Retired	45 (16.5%)
Other	3 (1.1%)
Missing	0
Time since diagnosis	
Less than 1 year	36 (13.2%)
1 to 3 years	72 (26.4%)
3 to 5 years	58 (21.2%)
5 to 10 years	65 (23.8%)
10 years or more	42 (15.4%)
Missing	0
Follow-up establishment	
Specialized cancer center	102 (37.6%)
Hospital center	93 (34.3%)
Clinic	65 (24.0%)
Private breast center	9 (3.3%)
Other	2 (0.7%)
Missing	2
Metastatic cancer on diagnosis	
Yes	119 (43.6%)
No	150 (54.9%)
Do not know	4 (1.5%)
Missing	0

Abbreviations: N = number.

**Table 2:** Oncologist demographics (N = 40).

Variable	N (%)
Gender	
Female	14 (35.0%)
Male	26 (65.0%)
Missing	0
Age	
Under 40 years	21 (52.5%)
40–49 years	7 (17.5%)
50–59 years	10 (25.0%)
60 years and over	2 (5.0%)
Missing	0
Practice establishment	
Hospital center	26 (65.0%)
Specialized cancer center	10 (25.0%)
Clinic	3 (7.5%)
Private breast center	1 (2.5%)
Missing	0
Percentage of patients with local HER2-positive cancer in the clinic	
Mean (SD)	50.1 (17.8)
Median	50.0
[Q1; Q3]	[40.0; 61.2]
Min; Max	5; 80
Missing	0
Percentage of patients with metastatic HER2-positive cancer in the clinic	
Mean (SD)	49.9 (17.8)
Median	50.0
[Q1; Q3]	[38.8; 60.0]
Min; Max	20; 95
Missing	0

Abbreviations: Max = maximum; Min = minimum; N = number; Q1 = 1st quartile (25th percentile); Q3 = 3rd quartile (75th percentile); and SD = standard deviation.

## Data Availability

The datasets used in the current study are available as Supplementary Information.

## Supplementary Materials

The following supporting information can be downloaded at https://www.mdpi.com/article/10.3390/cancers17081349/s1, File S1: Patient survey; File S2: Oncologist survey; File S3: Full dataset from patients; and File S4: Full dataset from oncologists.
