# Supplementary material for: HERmione: Understanding the Needs of Patients Living with Metastatic HER2-Positive Breast Cancer Through a Cross-Sectional Survey in Parallel with Patients and Oncologists
Source: Cancers (Basel). 2025 Apr 17;17(8):1349. doi: 10.3390/cancers17081349 (PMC12025477; doi:10.3390/cancers17081349)
Supplement: Supplementary file 1 [file cancers-17-01349-s001.zip › cancers-3456703 Supplementary File 1_patient questionnaire edited.pdf]

Supplementary File 1: Patient questionnaire (translated to English from original French).

Support needs of HER2+ patients

**Version**	V4 - French
**Drafting date**	08/06/2022
**Pathology(ies) concerned**	Metastatic HER2+ breast cancer
**Target number of respondents**	100 HER2+ metastatic patients in France
**Questionnaire structure**	1. Screener<br>2. Medical profile of patients<br>3. Living with and coping with the disease<br>4. Perceptions and experience of treatments<br>5. Quality of life
**Number of questions**	30 questions without the Quality of Life section—SF12<br>42 questions with the Quality of Life section—SF12

<u>Introductory message to Wefight patients</u>

Hello,

We invite you to take part in a study aimed at better understanding the management of patients living with HER2+ metastatic breast cancer**.**

Your participation in this survey is anonymous and confidential. The results of this study will be analyzed globally, and do not in any way permit the identification of participants.

These aggregated results will be shared with the Seagen laboratory and the Mon Réseau Cancer du Sein association.

The results of this survey are likely to be the subject of scientific publications.

In accordance with current legislation, you have the right to access, rectify and object to the processing of your data at any time during the survey.

To find out more about data processing and how to exercise your rights, please read our information and consent notice.

Your participation is voluntary. By clicking on "Start", you agree:

1. To volunteer to take part in the study

2. To give your explicit consent to the collection and processing of your personal health data as described in the downloadable notice.

You may withdraw your consent to the processing of your data in the context of this study at any time and without justification, without this affecting the legitimacy of the processing carried out prior to the withdrawal of your consent.

In accordance with Decree no. 2011-655 of June 10, 2011 on the procedures for reporting by patients or approved patient associations of adverse effects likely to be linked to the medicines and products mentioned in Article L. 5121-1 of the French Public Health Code, you, as a patient, are involved in pharmacovigilance. When you notice an adverse reaction to a medicine prescribed to you, you can send a report to the health authorities via the "signalement-sante.gouv.fr" website.

We thank you in advance for your participation.

The Wefight team.

This questionnaire will only take 10 minutes of your time.

PART 1: SCREENER

To begin this questionnaire, we're going to ask you a few questions about your personal situation.

To all

Q1. You are

Only one possible answer

1. A patient
2. A caregiver

To all

Q2. You are

Only one possible answer

1. A woman
2. A man

⇨ ***Stop if Q1=1 and Q2=2->*** *"This questionnaire is for patients only. Thank you for your interest in this survey."*

To all

Q3. What year were you born?

[Year field] (yyyy)

⇨ ***Stop if under 18, so if Q3>2004*** *-> "This questionnaire is for adults only. Thank you for your interest in this survey.*

To all

Q4. Are you currently...

Only one possible answer

1. Off work
2. Working part-time
3. Working full-time
4. Retired
5. Other, please specify

To all

Q5. What type of breast cancer are you/your loved one being treated for?

Breast cancer is HER2 positive when cancer cells overexpress HER2.

Only one possible answer

1. HER2-positive breast cancer (or tumors overexpressing HER2)
2. My / Her breast cancer is not HER2 positive→ Ask a few questions and say thank you
3. I don't know→ Ask a few questions and say thank you

To all

Q6. What is the stage of your / her cancer?

Only one possible answer

1. Localized (breast, axillary lymph nodes)→ Ask a few questions and say thank you
2. Metastatic (extension beyond the breast and axillary lymph nodes to other organs)
3. I don't know→ Ask a few questions and say thank you

To all

Q7. Where are you being treated?

Only one possible answer

1. A Cancer Research Center (CLCC)
2. A university hospital (CHU)
3. A regional hospital (CHR)
4. A hospital (CH)
5. A clinic
6. A private breast center
7. Other, please specify

To all

Q8. What kind of environment do you currently live in?

Only one possible answer

1. Rural (fewer than 2,000 inhabitants)
2. Small towns (2,000 to 19,999 inhabitants)
3. Medium-sized city (20,000 to 199,999 inhabitants)
4. Large city (100,000 inhabitants or more)
5. Greater Paris

PART 2: PATIENT MEDICAL PROFILE

We will now ask you a few questions about your diagnosis and the management of your cancer.

To all

Q9. When was your breast cancer diagnosed?

[Month/year field] (mm/yyyy)

To all

Q10. Was your cancer immediately metastatic (metastases in organs other than the breast at the time of breast cancer diagnosis)?

Please disregard lymph nodes in the armpit.

Only one possible answer

1. Yes
2. No
3. I don't know

To all

Q11. The drug treatments you are currently receiving for your cancer are taken in the form of...

Several answers possible

1. Injection at home
2. From injection to hospital
3. From tablet / capsule to take at home

To all

Q12. In addition to your oncologist, are you accompanied by a specialist in your care?

Only one possible answer

1. A coordinating nurse
2. An advanced practice nurse
3. A private nurse
4. I'm not accompanied by a nurse

To all

Q13. Would you say that, in the context of monitoring your illness, you feel... / In the context of monitoring your loved one's illness, you feel...

Only one possible answer

1. That it's easy to call on the care team when you need it, you feel well accompanied / when your loved one needs it, she feels well accompanied
2. It's difficult to call on the care team when you need it, you feel isolated / when your loved one needs it, she feels isolated

To all

Q14. Do you currently have someone close to you who regularly supports you through your illness?

Only one possible answer

1. Yes
2. No

If Q14=1

Q15. How does your loved one help you with your illness / How do you help your loved one with his or her illness?

Multiple answers possible

1. Accompanying you to medical appointments
2. Help with preparing my medical appointments
3. Help with treatment monitoring
4. Help with daily living
5. Help with administrative formalities
6. Moral and psychological support
7. Other, please specify

PART 3: LIVING WITH AND COPING WITH ILLNESS

To all

Q16. Which of the following statements applies to you at the moment?

Only one possible answer

1. I feel well informed about my illness
2. I don't think I have all the information about my disease and I'd like to know more.
3. I don't think I have all the information about my illness, but I rely completely on the care team.

To all

Q17. Would you say that you would be able to easily explain your illness to your loved ones? / is your loved one able to easily explain his or her illness to you?

Only one possible answer

1. Yes, we do.
2. Yes, rather
3. No, rather not
4. No, not at all

To all

Q18. Which of the following aspects of your illness do you need information about? / Which of the following aspects of your loved one's illness do you need information about?

Several answers possible

1. Current treatments
2. New treatments available
3. Advances in clinical research and therapeutic trials
4. Undesirable effects
5. The prognosis
6. The particularities of HER2-positive breast cancer compared to other cancers
7. The particularities of metastatic breast cancer
8. The risk of relapse
9. Alternative and complementary medicine (hypnosis, acupuncture, dietary supplements, etc.)
10. Other (please specify)

To all

Q19. Apart from your hospital care team, what means do you use to seek information about your illness/their illness?

Several answers possible

1. A patient association
2. Other patients in the same situation
3. A healthcare professional (pharmacist, general practitioner, etc.)
4. Media (press, television, radio, magazines)
5. Specialized health websites (including discussion forums)
6. Social networks
7. Healthcare applications dedicated to your care pathway
8. Someone I know (family, friends...)
9. Other, please specify
10. I'm not looking for information about my illness

To all

Q20. Have you been offered one or more of the following supportive care options since you were diagnosed with metastatic disease?

Oncology support care" is defined as **all the care and support** *required by patients throughout their illness. It is carried out in association with any specific cancer treatments that may be put in place.* ***It includes psychological support, pain management, adapted nutrition, physical activity, management of sexual dysfunction, etc.***

Multiple answers possible

1. Consultation with a pain management specialist
2. Consultation with a psychologist/psychiatrist
3. Practice a physical activity adapted to my illness with professionals
4. Consultation with a dietician or nutrition specialist
5. Consultation for disease-related sexual problems
6. Care by a social worker
7. Socio-aesthetics (beautician...)
8. Other, please specify
9. I did not use supportive care

To all

Q21. Would you say that in the face of illness, you currently feel... / your loved one currently feels...

Multiple answers possible

1. Confident
2. Combative
3. Optimistic
4. Surrounded
5. Determined
6. Resigned
7. Only
8. Pessimist
9. Anxious, depressed
10. Powerless

To all

Q22. What are the main difficulties you have encountered since being diagnosed with metastatic disease?

Several answers possible

1. Treatment side effects
2. The impact on my professional life
3. Pain management
4. Illness-related costs
5. Fatigue
6. Relationships with those closest to me (family, friends...)
7. Relations with care teams
8. Administrative management of the disease
9. Libido problems
10. Intellectual difficulties (memory, concentration, etc.)
11. Psychological difficulties in coping with illness and treatment (anxiety, depression...).
12. Other, please specify

To all

Q23. Did you feel that your opinion was taken into account when the last treatment you received was initiated?

Only one possible answer

1. Yes, I shared my wishes and expectations regarding my treatment with the care team and they took them into account.
2. Yes, I have expressed my wishes and expectations regarding my treatment, but they have not been taken into account.
3. No, I put all my trust in the care team in charge of my treatment.

To all

Q24. Do you keep up to date on new treatments for HER2-positive breast cancer?

Only one possible answer

1. Yes
2. No, because today I don't know where to find this information.
3. No, even if I know where to find this information

To all

Q25. In your opinion, access to these innovative treatments for patients in France is ...

Only one possible answer

1. Very easy
2. Pretty easy
3. Rather difficult
4. Very difficult
5. I don't know

PART 4: PERCEPTIONS AND EXPERIENCE OF TREATMENTS

We're now going to talk more specifically about your experience of cancer treatment.

To all

Q26. Which side effects related to your current treatment are the most difficult to manage on a daily basis / to your loved one's current treatment are the most difficult to manage on a daily basis?

Multiple answers possible

1. Fatigue, drowsiness
2. Sleep disorders
3. Memory disorders
4. Headaches, dizziness
5. Muscle and joint pain
6. Diarrhea
7. Constipation
8. Loss of appetite
9. Hair loss
10. Nausea, vomiting
11. Weight change (gain or loss)
12. Libido disorders
13. Tingling or loss of feeling in fingers and toes
14. Dryness, rashes
15. Mucositis (mouth ulcers, mouth lesions...)
16. Hematological disorders (anemia, decrease in white blood cells, platelets, etc.)
17. Other
18. None of the above

To all

Q27. In your opinion, the use of oral treatments (capsule/tablet) specific to your metastatic breast cancer presents...

Only one possible answer

1. More advantages than the systemic route (intravenous and subcutaneous)
2. More drawbacks than the systemic route (intravenous and subcutaneous)

If Q27=1

Q28. What do you think are the main advantages of using <u>oral treatments</u> (capsules/tablets) specific to your breast cancer? Oral anti-cancer treatments are...

Several answers possible

1. Efficient
2. Everyday practice
3. Well tolerated (few side effects)
4. Facilitate autonomous treatment
5. Little impact on my daily activities
6. Longer-lasting action
7. Act faster
8. Prolong the effects of chemotherapy
9. Avoid too many trips to healthcare facilities
10. Make it easier to adapt doses to my particular case
11. Cutting-edge, modern

12. Easily available from your local pharmacy
13. Do not require venous access (catheter, PAC)
14. None of the above

If Q27=2

Q29. What do you think are the main disadvantages of using oral treatments (capsules/tablets) specific to your breast cancer? Oral anti-cancer treatments are...

Multiple answers possible

1. Less effective than intravenous treatments
2. Difficult to take on a daily basis
3. Poorly tolerated (side effects)
4. Make autonomous treatment difficult
5. Strong impact on my daily activities
6. Shorter acting time
7. Act less quickly
8. Reduce interaction with specialized care teams
9. None of the above

To all

Q30. And in your opinion, what would be important to put in place to help patients take their oral anti-cancer treatment (tablet/capsule) at home?

Multiple answers possible

1. An alert on the phone (application, message)
2. A website providing information on the adverse effects of tablet medications
3. A printed user guide
4. Explanatory tutorial videos
5. A treatment-specific pillbox
6. A follow-up booklet
7. Access to therapeutic education programs in healthcare establishments
8. Access to oral therapy follow-up support programs (specialized platform, health application, etc.)
9. A home visit from a nurse
10. Other, please specify

PART 5: QUALITY OF LIFE

We would now like to assess your quality of life with the disease.

SF-12v2® Health Survey © 1993, 2003, 2012, 2013
Medical Outcomes Trust and
QualityMetric Incorporated.

All rights reserved.

SF-12® is a registered trademark of
Medical Outcomes Trust.
(SF-12v2® Health Survey Standard,
France (French))

Your health and well-being

The following questions are about your health, as you feel it. This information will help us to better understand how you feel in your everyday life. Thank you for completing this questionnaire!

For each of the following questions, select the option that best matches your answer.

Overall, do you think your health is:

Excellent
Very good
Good
Poor
Wrong

Here's a list of activities you may need to do in your daily life.

For each of them, indicate whether you are limited by your current state of health.

Indicate whether your current state of health limits your ability to perform moderate physical effort, such as moving a table, vacuuming or playing bowls.

Yes, very limited
Yes, a bit limited
No, not limited at all

Indicate whether your current state of health limits your ability to climb several flights of stairs.

Yes, very limited
Yes, a bit limited

No, not limited at all

In the last 4 weeks, have you had any of the following problems at work or with other regular daily activities, due to your physical condition?

In the last 4 weeks, have you accomplished less than you would have liked because of your physical condition?

All the time
Very often
Sometimes
Rarely
$
Never

In the last 4 weeks, have you had to stop doing certain things at work or other usual activities because of your physical condition?

All the time
Very often
Sometimes
Rarely
Never

In the last 4 weeks, and due to your emotional state (such as feeling sad, nervous or depressed), have you had the following problems at work or during other regular daily activities?

Over the past 4 weeks, and because of your emotional state (such as feeling sad, nervous or depressed), have you accomplished less than you would have liked?

All the time
Very often
Sometimes
Rarely
Never

Over the past 4 weeks, and due to your emotional state (such as feeling sad, nervous or depressed), have you worked or had other regular daily activities less attentively than usual?

All the time
Very often
Sometimes
Rarely
Never

Over the past 4 weeks, to what extent has your physical pain limited your work or domestic activities?

Not at all<br>A little bit<br>Medium<br>Many<br>Enormously
The following questions ask how you've been feeling over the past 4 weeks. For each question, please indicate the answer that seems most appropriate to you.
In the last 4 weeks, have there been times when you've felt calm and relaxed?<br><br>All the time<br>Very often<br>Sometimes<br>Rarely<br>Never
In the last 4 weeks, have there been times when you've felt bursting with energy?<br><br>All the time<br>Very often<br>Sometimes<br>Rarely<br>Never<br><br>In the last 4 weeks, have there been times when you've felt sad or depressed?<br><br>All the time<br>Very often<br>Sometimes<br>Rarely<br>Never
In the last 4 weeks, have there been times when your health, either physical or emotional, has interfered with your social life and your relationships with others, your family, friends and acquaintances?<br><br>All the time<br>Very often<br>Sometimes<br>Rarely<br>Never

END PAGE :

The questionnaire is now closed.
Thank you for your participation.
