# Supplementary material for: HERmione: Understanding the Needs of Patients Living with Metastatic HER2-Positive Breast Cancer Through a Cross-Sectional Survey in Parallel with Patients and Oncologists
Source: Cancers (Basel). 2025 Apr 17;17(8):1349. doi: 10.3390/cancers17081349 (PMC12025477; doi:10.3390/cancers17081349)
Supplement: Supplementary file 1 [file cancers-17-01349-s001.zip › cancers-3456703 Supplementary File 2_oncologist questionnaire edited.pdf]

Supplementary File 2: Oncologist questionnaire (translated to English from original French).

Needs and support for HER2+ patients

**Version**	V2
**Drafting date**	01/07/2022
**Pathology(ies) concerned**	Metastatic HER2+ breast cancer
**Type(s) of respondent(s)**	Healthcare professionals
**Target number of respondents**	40 oncologists in mainland France
**Questionnaire structure**	1. Socio-demographic profile of oncologists<br>2. Perception of the disease journey<br>3. Perception of treatment experience
**Number of questions**	26 questions

Hello,

We are currently conducting a study on the **care pathway of patients with HER2+ cancer** to better understand their experiences and expectations.

Please note that this study is not **intended for promotional purposes.** Similarly, any documents you may be presented with during the course of the study are intended to provide the study sponsor:

- A better understanding of your opinions,
- A better perception of your attitudes to medical issues.

All the documents you will see are **provided for exploratory purposes only and are not intended for promotional purposes.**

We would like to remind you of your obligations in terms of reporting any adverse reactions/ pharmacovigilance cases that may be mentioned during this market study.

As this is a market survey, **all your answers will of course be treated as confidential.** Nevertheless, should you raise an adverse reaction/pharmacovigilance case relating to a specific patient in the course of the questionnaire, we will be obliged to remind you of your obligation to report it, even if you have already reported it directly to the laboratory's pharmacovigilance department or to the relevant authorities**. In such a case, you will also be asked to agree to waive the confidentiality clause**, so that the adverse reaction/pharmacovigilance case can be reported to our customer. However, you may refuse to waive this clause if you wish.

To all

Do you agree to take part in this survey under such conditions?

Only one possible answer

1. Yes
2. No **= STOP INTER**

PART 1: SOCIO-DEMOGRAPHIC PROFILE OF ONCOLOGISTS

To all

Q1. You are

Only one possible answer

1. Oncologist
2. Other (please specify)→ STOP INTER: "This survey is aimed at oncologists. Thank you for your interest in this survey".

To all

Q2. You are

Only one possible answer

1. A woman
2. A man

To all

Q3. How old are you?

[Digital field - Terminal 18-99*]*

RECODE AGE

Under 40
40 to 49 years old
50 to 59 years
60 and over

To all

Q4. Which of the following best describes your practice?

Only one possible answer

1. Mostly self-employed (= more than 50% of working time)
2. Mostly hospital practice (= more than 50% of working time)
3. Mixed practice (50% hospital and 50% city)

To all

Q5. Where do you work?

Only one possible answer

A Cancer Research Center (CLCC)
University Hospital Center (CHU)
A regional hospital (CHR)
A hospital (CH)
A clinic
A private breast center
Other, please specify

To all

Q6. How much of your business is devoted to managing patients with HER2+ cancer?

Total must equal 100%.

____% of my activity is devoted to managing patients with HER2+ cancer?

→ STOP INTER if 0%: "This survey is aimed at oncologists who devote time to managing patients with HER2+ breast cancer. Thank you for your interest in this survey.

To all

Q7. Among these HER2+ patients, what percentage are...

Total must equal 100%.

1. In the early stages __%
2. Metastatic __%→ STOP INTER if item 2 = 0%: "This survey is aimed at oncologists following up patients with HER2+ metastatic breast cancer. Thank you for your interest in this survey."

To all

Q8. In your opinion, what percentage of patients with HER2+ metastatic cancer are accompanied by a nurse (coordination, advanced practice, private practice) as part of their follow-up?

Only one possible answer

1. ___ % of HER2+ metastatic patients
2. I don't know

PART 2: PERCEPTION OF THE DISEASE JOURNEY

Display the sentence below throughout the questionnaire, above each question:
This question concerns only patients with HER2+ metastatic cancer.

To all

Q9. Overall, would you say that the patient pathway for patients with HER2+ metastatic breast cancer currently in place at your facility is satisfactory?

Only one possible answer

1. Fully satisfactory
2. Quite satisfactory
3. Rather unsatisfactory
4. Not at all satisfactory

To all

Q10. What do you think could be done to improve the pathway for patients with HER2+ metastatic breast cancer at your facility?

(Open question)

____

To all

Q11. Would you say that, when it comes to monitoring their disease, these patients tend to feel...

Only one possible answer

1. It's easy for them to call on the care team when they need it, and they feel well cared for.
2. They find it difficult to call on the care team when they need it, and feel isolated.

To all

Q12. **Which of these statements would you say best describes your patients overall?**

Only one possible answer

1. They feel well informed about their illness
2. They don't have all the information about their disease and want to know more
3. They don't have all the information about their illness, but they rely completely on their care team.

To all

Q13. Which of the following aspects of HER2+ metastatic cancer do your patients need information on?

Several answers possible

1. Current treatments
2. New treatments available
3. Advances in clinical research and therapeutic trials
4. Undesirable effects
5. The prognosis
6. The particularities of HER2-positive breast cancer compared to other cancers
7. The particularities of metastatic breast cancer

8. The risk of relapse
9. Alternative and complementary medicine (hypnosis, acupuncture, dietary supplements, etc.)
10. Other (please specify)

To all

Q14. Do you offer the following support services to your patients with HER2+ metastatic cancer?

Several answers possible

1. Consultation with a pain management specialist
2. Consultation with a psychologist/psychiatrist
3. Practice a physical activity adapted to my illness with professionals
4. Consultation with a dietician or nutrition specialist
5. Consultation for disease-related sexual problems
6. Care by a social worker
7. Socio-aesthetics (beautician...)
8. Other, please specify
9. I do not offer supportive care

To all

Q15. Would you say that HER2+ cancer patients feel most at ease with their disease?

Several answers possible

1. Confident
2. Combatives
3. Optimists
4. Surroundings
5. Determined
6. Resigned
7. Only
8. Pessimists
9. Anxious, depressed
10. Powerless

To all

Q16. In your opinion, what are the main difficulties encountered by patients since the diagnosis of metastatic disease?

Several answers possible

1. Treatment side effects
2. The impact on their professional lives
3. Pain management
4. Illness-related costs
5. Fatigue
6. Relationships with those closest to me (family, friends...)
7. Relations with care teams
8. Administrative management of the disease
9. Libido problems
10. Intellectual difficulties (memory, concentration, etc.)
11. Psychological difficulties in coping with illness and treatment (anxiety, depression...).
12. Other, please specify

To all

Q17. In your opinion, involving patients in the choice of treatments is...

Only one possible answer

1. Essential
2. Important but not essential
3. Secondary

To all

Q18. Would you say that you involve patients in the choice of treatments at the time of initiation?

Only one possible answer

1. Yes, systematically
2. Yes, but not systematically
3. No

To all

Q19. Currently, would you say you are well informed or not about therapeutic innovations in HER2+ metastatic breast cancer?

Only one possible answer

1. Very well informed
2. Rather well-informed
3. Rather uninformed
4. Very uninformed

To all

Q20. Do you provide your patients with information on therapeutic innovations for HER2+ metastatic breast cancer?

Only one possible answer

1. Yes, often
2. Yes, sometimes
3. Yes, rarely
4. No, never

PART 4: PERCEPTION OF TREATMENT EXPERIENCE

We will now talk more specifically about your perception of the treatment experience of patients living with metastatic HER2+.

To all

Q21. What do you think are the most difficult treatment-related adverse events for patients with HER2+ metastatic cancer?

Multiple answers possible

1. Fatigue, drowsiness
2. Sleep disorders
3. Memory disorders
4. Headaches, dizziness
5. Muscle and joint pain
6. Diarrhea
7. Constipation
8. Loss of appetite
9. Hair loss
10. Nausea, vomiting
11. Weight change (gain or loss)
12. Libido disorders
13. Tingling or loss of feeling in fingers and toes
14. Dryness, rashes
15. Mucositis (mouth ulcers, mouth lesions...)
16. Hematological disorders (anemia, decrease in white blood cells, platelets, etc.)
17. Other
18. None of the above

To all

When you announce that your disease has progressed to the metastatic stage, who is in charge of explaining the new treatments and their associated side effects?

Only one possible answer

1. Just yourself
2. A nurse only, during a specific consultation
3. A pharmacist only, during a specific consultation
4. Several different professionals during a specific consultation (oncologist, pharmacist, nurse)
5. Other (please specify)

To all

Q22. In your opinion, for patients, the use of targeted therapies, oral anticancer treatments specific to these HER2+ metastatic breast cancers, presents...

Only one possible answer

1. More advantages than the systemic route (intravenous and subcutaneous)
2. More drawbacks than the systemic route (intravenous and subcutaneous)

To those who say that oral treatments offer more advantages

Q23. In your opinion, what are the main advantages perceived by patients to the use of targeted therapies, oral anticancer treatments specific to HER2+ metastatic breast cancer? Oral anticancer treatments are...

Several answers possible

1. Efficient
2. Everyday practice
3. Well tolerated (few side effects)
4. Facilitate autonomous treatment
5. Little impact on their daily activities
6. Longer-lasting action
7. Act faster
8. Prolong the effects of chemotherapy
9. Avoid too many trips to healthcare facilities
10. Make it easier to adapt doses to individual needs
11. Cutting-edge, modern
12. Easily available from your local pharmacy
13. Do not require venous access (catheter, PAC)
14. None of the above

To those who say that oral treatments have more disadvantages

Q24. What do you see as the main drawbacks to the use of targeted therapies, oral anticancer treatments specific to HER2+ metastatic breast cancer? Oral anticancer treatments are...

Several answers possible

1. Less effective than intravenous treatments
2. Difficult to take on a daily basis
3. Poorly tolerated (side effects)
4. Make autonomous treatment difficult
5. High impact on their daily activities
6. Shorter acting time
7. Act less quickly
8. Reduce interaction with specialized care teams
9. None of the above

To all

Q25. And in your opinion, what would be important to put in place to help patients take their oral anti-cancer treatment at home?

Several answers possible

1. An alert on the phone (application, message)
2. A website providing information on the adverse effects of tablet medications
3. A printed user guide
4. Explanatory tutorial videos
5. A treatment-specific pillbox
6. A follow-up booklet
7. Access to therapeutic education programs in healthcare establishments
8. Access to oral therapy follow-up support programs (specialized platform, health application, etc.)
9. A home visit from a nurse
10. Other, please specify

END PAGE :

The questionnaire is now closed.
Thank you for your participation.
